# Relações entre a Redução de Estrogênio, Obesidade e Insuficiência Cardíaca com Fração de Ejeção Preservada

**DOI:** 10.36660/abc.20200855

**Published:** 2021-09-24

**Authors:** Allan Kardec Nogueira de Alencar, Hao Wang, Gláucia Maria Moraes de Oliveira, Xuming Sun, Gisele Zapata-Sudo, Leanne Groban

**Affiliations:** 1 Faculdade de Medicina de Petrópolis Petrópolis RJ Brasil Faculdade de Medicina de Petrópolis, Petrópolis, RJ - Brasil; 2 Wake Forest School of Medicine Departments of Anesthesiology Winston-Salem North Carolina Estados Unidos da América Wake Forest School of Medicine - Departments of Anesthesiology, Winston-Salem, North Carolina - Estados Unidos da América; 3 Universidade Federal do Rio de Janeiro Departamento de Clínica Médica Faculdade de Medicina Rio de Janeiro RJ Brasil Universidade Federal do Rio de Janeiro - Departamento de Clínica Médica, Faculdade de Medicina, Rio de Janeiro, RJ - Brasil; 4 Universidade Federal do Rio de Janeiro Instituto de Ciências Biomédicas Rio de Janeiro RJ Brasil Universidade Federal do Rio de Janeiro - Instituto de Ciências Biomédicas, Rio de Janeiro, RJ - Brasil; 5 Universidade Federal do Rio de Janeiro Instituto de Cardiologia Edson Saad Faculdade de Medicina Rio de Janeiro RJ Brasil Universidade Federal do Rio de Janeiro - Instituto de Cardiologia Edson Saad, Faculdade de Medicina, Rio de Janeiro, RJ - Brasil; 6 Wake Forest School of Medicine Winston-Salem North Carolina Estados Unidos da América Wake Forest School of Medicine - Internal Medicine-Section of Molecular Medicine, Winston-Salem, North Carolina - Estados Unidos da América

**Keywords:** Estrogênio, Obesidade, Insuficiência Cardíaca, Volume Sistólico, Menopausa, Adiposidade, Sobrepeso, Ecocardiografia/métodos, Índice de Massa Corporal

## Abstract

A prevalência de obesidade e insuficiência cardíaca com fração de ejeção preservada (ICFEP) aumenta significativamente em mulheres na pós-menopausa. Embora a obesidade seja um fator de risco para disfunção diastólica do ventrículo esquerdo (DDFVE), o mecanismo que liga a interrupção da produção de hormônios ovarianos, especialmente o estrogênio, ao desenvolvimento da obesidade, DDFVE, e ICFEP em mulheres em processo de envelhecimento não é claro. Estudos clínicos e epidemiológicos demonstram que mulheres na pós-menopausa com obesidade abdominal (definida pela circunferência de cintura) têm risco maior de desenvolver a ICFEP do que homens ou mulheres sem obesidade abdominal. Este estudo analisa dados clínicos que corroboram a existência de uma ligação de mecanismo entre a perda de estrogênio mais obesidade e o remodelamento ventricular esquerdo com ICFEP. Ele também discute os possíveis mecanismos celulares e moleculares para a proteção mediada por estrogênio contra tipos de células, depósitos de tecidos, função e metabolismo de adipócitos negativos que podem contribuir para a DDFVE e a ICFEP.

## Introdução

A prevalência da obesidade está aumentando constantemente em todo o mundo.^1^ Como a obesidade está associada à mortalidade alta e ao desenvolvimento de comorbidades, incluindo diabetes mellitus e doenças cardiovasculares (DCV), ela é um dos problemas de saúde pública mais difíceis enfrentados por nossa sociedade. Esse grupo de comorbidades relacionadas à obesidade, direta ou indiretamente (por exemplo, efeito colateral de quimioterapia com antraciclinas)^2^ frequentemente culmina em insuficiência cardíaca (IC).^3 - 6^

Apesar de a obesidade, definida como um índice de massa corporal (IMC) >30 kg/m^2^, é um preditor independente de IC incidente na população geral, há evidências de que o próprio sobrepeso (IMC 25-29 kg/m^2^) representa um aumento no risco de IC.^7 - 10^

Vários estudos mostram que medidas de adiposidade, tais como circunferência de cintura (CC), são melhores que medidas de adiposidade global, tais como peso e IMC, para se estimar o risco de DCV.^11 - 16^ A CC é independentemente associada à disfunção diastólica do ventrículo esquerdo (DDFVE), definida por parâmetros ecocardiográficos.^17^ Tanto a DDFVE quanto a obesidade são fatores comuns que contribuem para o aparecimento de insuficiência cardíaca com fenótipo de fração de ejeção preservada (ICFEP), e parecem ter uma ligação causal.^17 - 19^ Em um breve resumo, pacientes com IC pode ter fenótipos diferentes, de acordo com as características morfofuncionais da doença.^20^ Em resumo, os pacientes de IC são classificados de acordo com a função VE; aqueles com frações de ejeção VE menor ou igual a 40% se encaixam na categoria de insuficiência cardíaca com fração de ejeção reduzida (ICFER), enquanto os pacientes com frações de ejeção maiores ou iguais a 50% são considerados portadores de ICFEP. De acordo com diretrizes do *American College of Cardiology* (Colégio Americano de Cardiologia) e da *American Heart Association* (Associação Americana do Coração)^21^ também existe um grupo de pacientes intermediários ou limítrofes, que têm frações de ejeção entre 41 e 49%, às vezes chamados de ICFEI. Além disso, um subconjunto de pacientes com frações de ejeção acima de 40%, com ICFEP que tiverem ICFER anteriormente é considerado clinicamente diferente daqueles com frações de ejeção persistentemente preservadas ou reduzidas. Esta análise concentrou-se apenas na ICFEP, e, especificamente, as características do fenótipo do portador de ICFEP “obeso, do sexo feminino e fadigado”.^21^

Uma revisão da literatura de todos os fenótipos clínicos de ICFEP de referência para o leitor é a realizada por Silverman.^20^ Independentemente do fenótipo biológico, a ICFEP é uma síndrome clínica heterogênea, que inclui mecanismos fisiopatológicos de cardiomiócitos, de matriz extracelular, vasculares e relacionados a comorbidade.^22^ Ela é caracterizadas pela redução do volume diastólico final, hipertrofia do ventrículo esquerdo, e aumento do volume atrial esquerdo e da pressão de enchimento do ventrículo esquerdo. Essas anormalidades fisiopatológicas estão associadas ao aumento da rigidez do ventrículo esquerdo, à diminuição do relaxamento do ventrículo esquerdo, à hipertrofia de cardiomiócitos, à fibrose miocárdica intersticial, e à redução de capilares intramiocárdicos.^23 - 26^

Outro fator importante envolvido no fenótipo de ICFEP é o sexo. A ICFEP afeta desproporcionalmente mais mulheres (razão de sexos de aproximadamente 2:1) que homens.^27 , 28^ A prevalência mais alta de ICFEP em mulheres idosas^29^ parece estar relacionada à perda de hormônios ovarianos, principalmente estrogênios, que ocorre após a menopausa.

Portanto, esta revisão explora dados pré-clínicos e clínicos sobre a relação entre sexo, “gordura”, incluindo mecanismos de disfunção cardíaca causada por obesidade, especificamente a DDFVE e a ICFEP, e os efeitos cardioprotetores do estrogênio no metabolismo de gordura nas mulheres.

### Associação entre “gordura”, sexo e ICFEP: evidências clínicas

A IC é um problema grave cujo escopo tem aumentado. Apesar dos avanços terapêuticos recentes, a morbidade e a mortalidade após o aparecimento da IC ainda continuam significativas.^30^ Consequentemente, a prevenção da IC pela identificação das fases pré-clínicas da doença e gestão dos fatores de risco é uma prioridade. Considerando-se que 50 por cento de todos os pacientes com IC têm ICFEP,^31^ a fisiopatologia complexa dessa doença ainda não é completamente entendida, sem terapia específica disponível para melhorar os resultados para o paciente. Nesse contexto, vários estudos avaliaram a obesidade como um fator de risco para remodelamento do VE e ICFEP subsequente.^32 - 34^ Nesses estudos, a obesidade foi consistentemente associada à rigidez do VE e à disfunção diastólica, especialmente em mulheres.^17 , 35^

Um estudo comunitário clínico de 377 participantes, acima de 16 anos de idade, selecionados aleatoriamente avaliou a contribuição independente dos índices de adiposidade para as variações na velocidade transmitral (atrial) de inicial a final (E/A), conforme determinada pelo ecocardiograma. O principal objetivo foi esclarecer por que alguns estudo não conseguiram estabelecer uma contribuição da obesidade para a função diastólica do VE, enquanto outros demonstraram uma contribuição relativamente pequena. Para cada participante do estudo, foi determinada a relação independente entre adiposidade e funções das câmaras diastólica (E/A) ou sistólica (fração de ejeção do VE, FEVE), utilizando-se análise de regressão linear multivariada, com padronização para idade, gênero, aferições convencionais de pressão arterial diastólica ou sistólica, e ou o índice de massa do VE ou a espessura relativa da parede (calculada por ecocardiograma). A adiposidade central (CC) excessiva, mas o IMC não elevado, foi independentemente e inversamente correlacionada à E/A; os pesquisadores enfatizaram que a CC pode representar uma condição pré-clínica progressiva que contribui para a IC diastólica causada por obesidade.^36^ A CC só foi superada pela idade e se igualou à pressão arterial em magnitude do efeito sobre a E/A. Achados deste estudo também sugerem que, no nível populacional, a massa e a geometria do VE tem pouca ou nenhuma influência na patogênese das anormalidades diastólicas do VE causadas por obesidade.^36^ É interessante observar que não há relação entre CC e FEVE (disfunção sistólica), confirmando os achados de outros pesquisadores que identificaram a CC como fator de risco para ICFEP.^7 , 9 , 17 , 37 - 43^ Por último, é relevante observar que os dados relatados por esses autores foram restritos a mulheres, já que foi recrutada uma proporção limitada de participantes do sexo masculino.^36^ Dados adicionais do estudo clínico realizado por Canepa et al.,^17^ em que a amostra, para ambos os sexos, é parte do *Baltimore Longitudinal Study of Aging* (BLSA - Estudo Longitudinal de Envelhecimento de Baltimore) propõem que uma possível explicação fisiopatológica para a associação entre adiposidade e resultados cardiovasculares piores é sua relação com a DDFVE. Eles identificaram que a adiposidade central estava fortemente associada à disfunção do VE, particularmente com deficiência do relaxamento do VE. Os pesquisadores também identificaram que o efeito da adiposidade central sobre a DDFVE foi independente da adiposidade geral e, surpreendentemente, isso era mais pronunciado em homens que em mulheres. O efeito específico do gênero do acúmulo central de gordura na DDFVE foi classificado de acordo com parâmetros ecocardiográficos. O estudo confirmou que os relatórios anteriores demonstrando correlação negativa entre os índices de adiposidade e a relação E/A; entretanto, os autores também identificaram que a E/A sozinha não era suficiente para diferenciar entre sujeitos com função diastólica normal ou anormal, e as velocidades de fluxo mitral também foram significativamente afetados pelo aumento de pré-carga, uma condição frequentemente encontrada em sujeitos obesos. Quando esses tecidos foram padronizados pela combinação de medidas histológicas por Doppler da velocidade do anel mitral, ou parâmetros e’ ou dinâmicos (E/A), identificou-se que a relação E/e’ estava correlacionada positivamente à CC. Portanto, esse estudo epidemiológico ofereceu mais evidências da ligação entre obesidade central (CC) e a prevalência e o desenvolvimento de DDFVE, e de que essa ligação é influenciada pelo sexo.^17^

Uma limitação de estudos transversais é que eles apresentam um instantâneo de um momento único no tempo e, portanto, não conseguem capturar mudanças que ocorrem ao longo do tempo. As relações complexas entre envelhecimento, sexo, adiposidade, e mecanismos ventriculares foram avaliadas em um grande estudo longitudinal realizado em um período de 4 anos, em 1.402 sujeitos, com 45 anos de idade ou mais, que foram selecionados aleatoriamente de uma população de uma comunidade.^35^ Identificou-se que o ganho de peso durante o período de 4 anos estava associado a aumentos significativos da rigidez diastólica do VE, em homens e mulheres, porém era mais pronunciado em mulheres, indicando que há uma diferença entre os sexos em relação à biologia da rigidez ventricular relacionada à idade. Além disso, a avaliação da obesidade central em mulheres pode ajudar a identificar um grupo em risco mais alto da incidência de ICFEP, que pode se beneficiar de tratamento preventivo.^35^ Finalmente, os resultados deste estudo longitudinal confirmaram os achados das investigações transversais relacionadas à relação positiva entre CC e medidas ecocardiográficas da disfunção diastólica (por exemplo, relação E/e’).

É importante considerar como as alterações do depósito de tecido adiposo podem influenciar a ligação entre obesidade e deficiências da função e remodelamento cardíaca, especialmente entre mulheres. Apesar de se acreditar que os hormônios sexuais femininos causam o acúmulo de gordura nas nádegas, coxas e quadris de mulheres, o que pode ser essencial para fins de reprodução normal, as mudanças na distribuição da gordura corporal relacionadas à menopausa pode explicar parcialmente o aumento do risco de doenças cardiovasculares e metabólicas durante os anos pós-menopausa.^44 - 46^ Em 2011, Wehr et al.,^47^ publicaram os resultados de um estudo longitudinal das diferenças específicas associadas ao sexo na relação entre o produto de acumulação lipídica, que é calculado a partir da CC, e a mortalidade cardiovascular, bem como a presença de diabetes tipo 2. O estudo incluiu 2.279 homens e 875 mulheres na pós-menopausa, com um acompanhamento médio aos 77 anos. Os níveis de produto de acumulação lipídica foram associados à mortalidade por IC congestiva em todas as mulheres na pós-menopausa, e com a mortalidade global em mulheres diabéticas na pós-menopausa, mas não em homens. Esses dados não só corroboram o conceito de que a redistribuição da gordura após a perda de estrogênio pode contribuir para o avanço de doenças cardiovasculares, mas também identifica um biomarcador de risco simples e acessível, o produto de acumulação lipídica, que poderia identificar mulheres na pós-menopausa com risco cardiovascular mais alto.^47^

### Modalidades de tratamento para perda de peso: uma abordagem baseada em evidências para medir resultados em pacientes com ICFEP

Considerando a evidência clínica de interferência entre coração e “gordura”, em relação a ICFEP específica do sexo feminino, a redução do peso, ou a manutenção do peso corporal ideal é uma abordagem preventiva para mitigar alterações na estrutura e na função ventricular relacionadas à idade e à perda de estrogênio. Tratamentos essenciais para redução de peso incluem alterações nos hábitos alimentares passando para dietas com redução de caloria e gordura, aumento da atividade física ou exercício, e outras estratégias de modificação comportamental, tais como o automonitoramento (por exemplo, registro diário do consumo alimentar e da atividade física), e solução de problemas (por exemplo, identificação de barreiras e maneiras de superá-las). Além disso, a cirurgia bariátrica é outra estratégia eficiente para tratar pacientes gravemente obesos. Portanto, é importante fazer a revisão da literatura que aborda os efeitos de várias estratégias de perda de peso sobre os efeitos cardiovasculares em pacientes com DDFVE e ICFEP.

Estudos observacionais sugerem que pacientes com ICFEP que têm sobrepeso ou com obesidade de leve a moderada têm uma sobrevida mais longa do que os que têm peso normal.^18 , 48^ Entretanto, Kitzman et al.,^49^ relataram recentemente que 20 semanas de restrição calórica combinada com exercícios aeróbicos entre pacientes obesos mais velhos com ICFEP reduziram seu peso corporal e resultaram em melhorias na capacidade de exercício, definida pelo VO2pico. Além disso, a restrição calórica sozinha levou à diminuição da massa do VE e da espessura relativa da parede do VE, bem como deu um indício de melhorias na função diastólica, conforme o aumento de E/A observado nesse braço do tratamento, sem afetar a função cardíaca em repouso, ilustrado pela fração de ejeção ou pelo débito cardíaco derivado de Doppler.^49^ Relatou-se posteriormente que não foram observadas alterações em medidas de imagens por ressonância magnética (IRM) da gordura epicárdica ou pericárdica durante o tratamento, mas houve reduções significativas nos depósitos de gordura subcutânea das coxas e abdominal e gordura visceral, apenas no grupo que fez a dieta.^49^ Apesar de esses achados identificarem serem favoráveis à adoção de estratégias de redução de peso, juntamente com exercícios, para melhorar os prejuízos à capacidade de exercício e o consumo máximo de oxigênio associado à ICFEP entre pacientes obesos, eles também corroboram o conceito de que mecanismos extracardíacos estão envolvidos de maneira exclusiva na patogênese da ICFEP.^50 , 51^

Outra estratégia de redução de peso que leva à avaliação da ligação entre “gordura”, DDFVE e ICFEP é a cirurgia bariátrica. Na realidade, pacientes com obesidade mórbida normalmente apresentam características hemodinâmicas e morfométricas cardíacas, tais como, elevação da pré-carga e da pós-carga cardíaca e aumento da câmara do VE e dimensões da parede do VE, que contribuem para a rigidez miocárdica e deficiências no relaxamento miocárdico.^52^ Vários estudos clínicos^52 - 55^ e uma revisão sistemática com meta-análise^56^ relataram os benefícios da cirurgia bariátrica com perda de peso subsequente nas medidas ecocardiográficas e de IRM da estrutura do VE, incluindo reduções significativas da espessura da parede e da massa do VE, e da função diastólica. Junto com as melhorias da função diastólica, outros estudos identificaram que a grande perda de peso após a cirurgia bariátrica também resultou em mudanças favoráveis do metabolismo muscular^57^ e das características da elasticidade arterial.^58 , 59^

### Ligação entre adiposidade regional específica cardíaca, DDFVE e ICFEP

Além da gordura periférica e da gordura total que estão ligadas à DDFVE, o possível papel da adiposidade específica da região cardíaca (por exemplo, depósitos de gordura pericárdica e epicárdica) não deve ser ignorado.^60^ As gorduras pericárdica e epicárdica, geralmente encontrada em pacientes obesos e com sobrepeso, são consideradas depósitos adiposos ectópicos que levam a um estado lipotóxico em proximidade ao músculo cardíaco e às artérias coronárias.^61 , 62^ Além disso, sabe-se que a síndrome metabólica, uma doença comum entre paciente obesos e com sobrepeso,^63^ está associada ao aumento do volume de tecido adiposo próximo ao coração,^60^ especialmente o acúmulo de gordura epicárdica,^64^ e isso está significativamente ligado a eventos cardiovasculares adversos,^65 - 69^ incluindo a ICFEP.^61 , 70 , 71^ A correlação direta entre esses depósitos locais de gordura e a DDFVE pode ser explicada, em parte, por processos parácrinos, pelos quais as citocinas pró-inflamatórias e outros mediadores prejudiciais (por exemplo, TNF-α e IL-6),^72^ chamados coletivamente de adipocinas, são liberados dos repositórios adiposos locais.^61 , 72 - 74^

A distinção entre os dois depósitos de gordura e sua respectiva ligação à DDFVE pode ser importante anatômica e bioquimicamente. Por exemplo, a gordura epicárdica está localizada entre a parede externa do músculo cardíaco e a camada visceral do pericárdio,^64^ e sua proximidade ao miocárdio é significativa, pois ambas as camadas de tecido compartilham da mesma microcirculação sanguínea, as artérias coronárias.^64^ Possíveis interações podem ser elicitadas quando adipócitos disfuncionais de depósitos de gordura cardíaca liberam adipocinas pró-inflamatórias para a microcirculação,^37^ que, por sua vez, interagem com cardiomiócitos e fibroblastos cardíacos. Essas células respondem independentemente às adipocinas que contribuem para o processo patológico da fibrose miocárdica,^75^ levando portanto à remodelamento miocárdica, por processos fibróticos e inflamação de baixo grau, que podem intensificar a hipertrofia do VE, rigidez da parede, e avanço da DDFVE.^76 - 79^ A gordura pericárdica, que pode ser chamada mais especificamente de gordura paracárdica ou gordura intratorácica,^80^ é a gordura depositada fora do pericárdio parietal. Esse depósito de gordura de origina do mesênquima torácico primitivo e é alimentado por fontes não coronárias. Embora já se tenha relatado que os aumentos no volume de gordura paracárdica na ICFEP levem à uma carga mecânica do tipo compressiva no miocárdio, o que afeta o enchimento do VE,^81^ também se observaram processos parácrinos também foram observados. A adiposidade pericárdica excessiva contém altos níveis de mediadores pró-inflamatórios que, quando liberados dos adipócitos, promovem uma rotação de colágeno, levando à rigidez miocárdica, lusitropismo prejudicado e subsequente DDFVE.^82^ Realmente, Konishi et al.,^61^ relataram que um volume alto de gordura pericárdica estava relativamente correlacionado a aumentos derivados de Doppler na pressão de enchimento, ou E/e’, em pacientes com ICFEP. Além disso, estudos documentaram um forte potencial de a adiposidade epicárdica estar associada com o mau prognóstico em pacientes com DDFVE e ICFEP obesos e com sobrepeso.^71 , 83 - 85^

Considerando a ligação entre depósitos de gordura cardíaca local e a saúde cardiovascular adversa, as estratégias de redução de peso devem ser fortemente consideradas no arsenal terapêutico para a gestão de pacientes com DDFVE obesos. É interessante observar que em mulheres na pós-menopausa com ICFEP, Brinkley et al.,^86^ demonstraram que a restrição calórica, os exercícios aeróbicos ou uma combinação de tratamentos reduziram significativamente o peso corporal e a gordura pericárdica, e que as mudanças na gordura pericárdica estavam inversamente correlacionadas ao condicionamento cardiorrespiratório definido por VO_2_max. Com certeza, terapias futuras que têm como alvo processos inflamatórios de baixo grau resultantes de depósitos de gordura epicárdica e pericárdica também poderiam limitar o avanço da DDFVE.

### Ligação entre estrogênio - risco cardiovascular induzido por gordura

A alta prevalência da ICFEP entre mulheres mais velhas em comparação a homens mais velhos com IC é bem aceita.^27 , 28^ O papel que as diferenças na distribuição de adipócitos entre homens e mulheres pode ter em relação a esses diferenciais específicos do sexo na prevalência da IC é novo e ainda em avaliação. Realmente, as mulheres têm mais gordura corporal que os homens, mas, diferentemente das consequências metabólicas adversas da obesidade central que é típica de homens, a distribuição subcutânea da gordura corporal gluteofemoral ou em formato de pera de muitas mulheres está associada a um risco cardiometabólico mais baixo.^87 , 88^ Entretanto, com o avanço da idade, há uma mudança geral e expansão da gordura, do compartimento subcutâneo para o visceral.^87 - 89^ Em homens idosos, isso significa o aumento da adiposidade visceral abdominal, enquanto em mulheres idosas isso envolve uma redistribuição de gordura do compartimento subcutâneo gluteofemoral para o compartimento visceral-abdominal.^87 - 89^ Em ambos os casos, o risco de doença cardiovascular aumenta com a expansão de gordura visceral do compartimento abdominal relacionado à idade.^89 , 90^

Como apontado na introdução, a perda de hormônios gonadais em mulheres mais velhas parece representar um componente associado ao aumento do risco de desenvolvimento de ICFEP. Como as mulheres têm menor possibilidade de desenvolver DCV antes da menopausa,^91^ a produção de estrogênio ovariano parece proteger contra a IC.^92 , 93^ De forma consistente, há vários relatórios que confirma os efeitos benéficos do estrogênio no sistema cardiovascular.^94 - 96^

Para entender o papel específico dos hormônios gonadais na expansão da gordura visceral relacionada à idade em mulheres, e, ao mesmo tempo, sua possível influência na função diastólica, é necessário realizar uma análise breve dos hormônios gonadais, particularmente os estrogênios e seus receptores primeiramente. Os três estrogênios que ocorrem naturalmente em mulheres são a estrona (E1), o estradiol (E2), e o estriol (E3). Uma quarta forma de estrogênio, o estetrol (E4), é produzida apenas durante a gravidez. Todas essas formas diferentes de estrogênio são sintetizadas a partir de andrógenos.^97^ Para simplificar, será utilizado o termo estrogênio incluindo todas as formas.

O estrogênio se liga a vários receptores, incluindo os receptores de estrogênio nuclear clássicos (RE), REα, e REβ, e um receptor acoplado à proteína G, RAPG.^98^ Os RE sinalizam não só por meio da regulação de transcrição gênica “clássica”, com também pela ativação de um caminho de sinalização “não nuclear”.^94 , 99^ O acúmulo de achados já foi bem descrito e analisado na literatura relacionada aos papéis desencadeados pelos RE na manutenção da homeostase do sistema cardiovascular.^99 - 101^

O estrogênio regula diretamente as distribuições de adiposidade por meio de receptores de estrogênio. No estado pré-menopausa, a gordura subcutânea tem relativamente mais receptores de estrogênio e progesterona que receptores de andrógenos, enquanto a gordura visceral tem níveis mais altos de receptores de andrógenos.^102^ Com a menopausa, a queda do estrogênio faz com que os receptores de estrogênio na gordura subcutânea sejam inativados, enquanto os receptores de andrógenos na gordura visceral se torna relativamente ativada, contribuindo, portanto para a relação inversa entre níveis de estrogênio e gordura visceral.^103 , 104^ Da mesma forma, em modelos de roedores com déficit de estrogênio induzido por ovariectomia, o aumento do peso corporal se deve principalmente pelo aumento de gordura visceral.^105^ A proteção estrogênica pode ser vista na administração sistêmica de estrogênio em modelos ovariectomizados em que a distribuição de gordura corporal reflete à das contrapartidas com gônadas intactas.^106^

Os papéis específicos podem compensar os receptores de esteroides REα e REβ no contexto da gordura um ao outro. Em um estudo recente de Zidon et al.,^107^ identificou-se que ratos KO comα RE de gônadas intactas são 25% mais pesados, com redução do gasto energético em comparação com o tipo selvagem com gônadas intactas e os ratos KO com ERβ.^107^ Além disso, após a ovariectomia, os ratos αKO não apresentaram aumento de peso corporal ou apresentaram resistência à insulina mais pronunciada, enquanto os do tipo selvagem e os βKO apresentaram, sugerido que a perda de sinal por REα facilita a disfunção metabólica induzida por ovariectomia. Esses novos dados sugerem ainda que após a deficiência de estrogênio, os REβ podem mediar benefícios metabólicos de proteção.^107^ Isso contradiz relatórios pré-clínicos anteriores demonstrando que dois RE clássicos no tecido adiposo regulam a gordura reciprocamente.^108 - 110^ Apesar dessa discrepância, a ligação entre os polimorfismos na estrutura dos RE clássicos e no aumento de risco em mulheres na pós-menopausa corroboram o papel importante de isoformas de ER na regulação da adiposidade, na disfunção metabólica, e no risco cardiovascular.^111 - 113^

### Adipocinas derivadas da gordura e funções no risco de doenças cardiovasculares

A principal função da gordura marrom, localizada principalmente ao redor do pescoço e grandes vasos sanguíneos do tórax, é gerar calor “desacoplando” a cadeia de fosforilação oxidativa dentro das mitocôndrias.^114^ A gordura visceral branca (gordura abdominal) está envolvida principalmente em uma rede multidirecional de sinalização autócrina, parácrina e endócrina que interconecta órgãos e tecidos. É a gordura branca que participa principalmente da patogênese de doenças metabólicas, tais como o diabetes mellitus tipo 2, resistência à insulina, hipertensão, doença cardíaca coronária, acidente vascular cerebral, e IC.^17 , 115 , 116^ Atualmente, está bem estabelecido que o tecido adiposo é um órgão endócrino ativo que secreta fatores bioativos heterogêneos chamados adipocinas^115^ incluindo citocinas e quimiocinas, fatores vasoativos e de coagulação, reguladores do metabolismo lipoproteico, e proteínas como adiponectina e leptina.^115^

Na obesidade, o aumento da massa de tecido adiposo tem sido associada a uma desregulação da secreção de adipocinas e à inflamação de tecido relacionada, o que representa uma ligação patogênica entre obesidade e o desenvolvimento de doenças cardiometabólicas.^117^ Em indivíduos obesos, o tecido adiposo é infiltrado por macrófagos ativados e vários outros tipos de células inflamatórias, levando ao aumento da produção de adipocinas pró-inflamatórias, tais como TNF-α, IL-6, proteína quimiotática de monócitos (MCP-1), resistina, leptina, lipocalina-2, proteína transportadora de ácidos graxos de adipócitos (A-FABP), inibidor do ativador de plasminogênio tipo 1.^116^ Esses fatores inflamatórios são componentes-chave do “eixo adipo-cardiovascular”, que media as interferências entre tecido adiposo e o sistema cardiovascular.

Entre os vários depósitos adiposos, o tecido adiposo perivascular tem uma contribuição importante na inflamação vascular, devido à sua proximidade à parede do vaso sanguíneo e suas propriedades pró-inflamatórias pronunciadas. Citocinas/adipocinas pró-inflamatórias liberadas de outros depósitos de tecido adiposo, tais como a gordura abdominal subcutânea, podem contribuir ainda mais para a inflamação vascular, por meio de suas ações endócrinas.^116^ Esses achados explicam, em parte, porque a CC pode ser considerada um biomarcador substituto do risco de DCV.

A adiponectina é uma das adipocinas mais abundante secretadas pelos adipócitos, representando 0,01% do teor de proteína plasmática em seres humanos^118^ A produção de adiponectina a partir de adipócitos brancos, que tem efeitos benéficos na sensibilidade à insulina e na função cardiovascular, é notadamente reduzida em indivíduos obesos.^116 , 119 , 120^ Estudos epidemiológicos demonstram que níveis de adiponectina circulante baixos, especialmente a forma com alto peso molecular, é um fator de risco de diabetes tipo 2, hipertensão, aterosclerose e infarto do miocárdio.^116^

#### Adiponectina e receptores de adiponectina

A relação entre obesidade e DDFVE pode estar relacionada à adiponectina e aos receptores de adiponectina. A adiponectina é composta de 247 resíduos de aminoácidos, organizados em uma região hipervariável N-terminal seguidos de um domínio colagenoso conservado de 22 repetições de Gly-Xaa-Yaa e um domínio globular *C* -terminal tipo C1q.^119^ A adiponectina está presente no plasma humano e de ratos nas três principais formas oligoméricas.^119 , 121 , 122^ A forma monomérica nunca foi detectada em condições nativas. A unidade básica de adiponectina oligomérica é um homotrímero chamado adiponectina de baixo peso molecular (LMW).^119 , 123 , 124^ Duas subunidades do trímero de adiponectina estão conectadas por uma ligação dissulfeto via resíduos de cisteína do domínio do tipo colágeno, que forma um hexâmero chamado adiponectina de médio peso molecular (MMW). O hexâmero fornece a base para a formação da adiponectina de alto peso molecular (HMW) em forma de buquê, composta de 12-18 hexâmeros.

É necessário realizar modificações pós-translação na proteína adiponectina para a organização intracelular do complexo oligomérico de HMW nos adipócitos.^125^ Várias formas de adiponectina agem em alvos diferentes e têm funções biológicas distintas.^119^

Os dois principais receptores de adiponectina (AdipoRs), AdipoR1 e AdipoR2, são estruturalmente e funcionalmente diferentes dos receptores acoplados à proteína G clássicos.^126^ O AdipoR1 é expresso em todos os locais, enquanto o AdipoR2 é expresso mais abundantemente no fígado.^126^ Tanto o AdipoR1 quanto o AdipoR2 são expressos em células cardíacas,^127^ mas a função exata desses dois receptores no stress antioxidativo/nitrativo e nas ações anti-inflamatórias nos cardiomiócitos ainda não está clara.

Embora os adipócitos tenham a principal contribuição para a adiponectina plasmática, a adiponectina também é expressa em cardiomiócitos,^127^ e a adiponectina derivada de cardiomiócitos é biologicamente ativa na proteção das células contra lesões isquêmicas via ativação parácrina/autócrina dos AdipoRs em ratos.^128^ Em pacientes com cardiomiopatia dilatada, a expressão da adiponectina cardíaca é diminuída.^129^

#### Adiponectina e DDFVE

Além dos AdipoRs, sugere-se também que a T-caderina também seja um receptor possível para a adiponectina^130^ e ele é altamente expresso no coração, no músculo liso, e no endotélio, representando o principal alvo da adiponectina no sistema cardiovascular.^131 , 132^ A T-caderina é ancorada na superfície da célula pelo glicosilfosfatidilinositol e desempenha um papel essencial na proteção cardíaca induzida por adiponectina em ratos^133^ agindo como um receptor de ligação de adiponectina fisiológico que permite a associação dessa adipocina ao tecido cardíaco.^133^

Como baixos níveis de adiponectina foram associados a complicações cardiometabólicas relacionadas à obesidade, sua função na manutenção da saúde cardíaca não deve ser ignorada^120 , 134 , 135^ Dados pré-clínicos mostram que a adiponectina pode atenuar ou evitar o avanço da DDFVE para ICFEP.^120 , 136 , 137^ Em um modelo com ratos de ICFEP induzida por aldosterona, Sam et al.,^137^ demonstraram que a falta de adiponectina estava associada ao aumento da pressão arterial sistólica, remodelamento do VE, disfunção diastólica, e congestão pulmonar. Já a hiperadiponectinemia em ratos transgênicos com superexpressão de adiponectina, relatada Tanaka et al.,^120^ amenizou a hipertrofia do VE induzida por aldosterona, a disfunção diastólica, e a congestão pulmonar, independentemente das mudanças na pressão arterial. A relação de enchimento inicial e descida inicial do anel mitral, ou E/e’, que foi aumentada nos ratos com ICFEP induzida por aldosterona e indica pressões de enchimento elevadas,^137^ foi significativamente atenuada em ratos transgênicos por adiponectina. Tanaka et al.,^120^ também detectaram que a superexpressão de adiponectina diminuiu o stress oxidativo e o manejo de cálcio, preservando a fosforilação de fosfolambano dependente da proteína quinase A (PKA). Além disso, a reposição de adiponectina em ratos knockout atenuou os índices de pseudonormalização transmitral de Doppler, que indicam conformidade com VE deficiente.^120^ Considerados em conjunto, esses achados pré-clínicos sugerem que a adiponectina possa ter um potencial terapêutico no controle da DDFVE e da ICFEP, por meio de ação direta no coração.

A observação deste estudo de que a adiponectina circulante não estava associada ao encurtamento fracional do VE é consistente com a literatura existente que sugere que a adiponectina age principalmente como um inibidor de hipertrofia cardíaca.

Estudos clínicos também corroboram a hipótese de associação entre adiponectina e função e estrutura cardíacas.^129 , 138^ Em um estudo coorte em uma comunidade, McMannus et al.,^139^ demonstraram que níveis plasmáticos relativamente mais altos de adiponectina associados à redução da massa do VE, sugerindo um efeito de proteção cardíaca. Existem dados clínicos adicionais que tratam das funções de proteção cardíacas da adiponectina no coração.^140 - 142^ Por exemplo, Francisco et al.,^143^ realizou uma revisão abrangente da relevância da sinalização por adiponectina na prevenção de disfunção diastólica relacionada à obesidade com ênfase em sua função de limitar a hipertrofia miocárdica, a fibrose cardíaca, o stress oxidativo e nitrativo, a aterosclerose, e a inflamação.

Como existem vários estudos mostrando o papel modulador da adiponectina na manutenção da função diastólica, vale mencionar que outros pesquisadores não relataram nenhuma relação com DDFVE associada à gordura. Sawada et al.,^144^ identificaram que, enquanto os níveis de adiponectina diminuíram significativamente com o aumento de adiposidade visceral na população em geral, a associação entre adiponectina e função diastólica não se deu independentemente da gordura. Em outras palavras, a diminuição dos níveis de adiponectina circulante não parece ter tido um papel central na associação entre adiposidade visceral e DDFVE. Como esse estudo foi realizado em uma população não portadora ou com grau 1 de DDFVE, os sujeitos com DDFVE mais moderada não foram incluídos. Esses autores sugeriram que um estudo de larga escala, incluindo pacientes com DDFVE moderada ou grave é necessário, para confirmar os achados.

## Diferenças de sexo, adiponectina e função cardíaca

**Figura 1:**
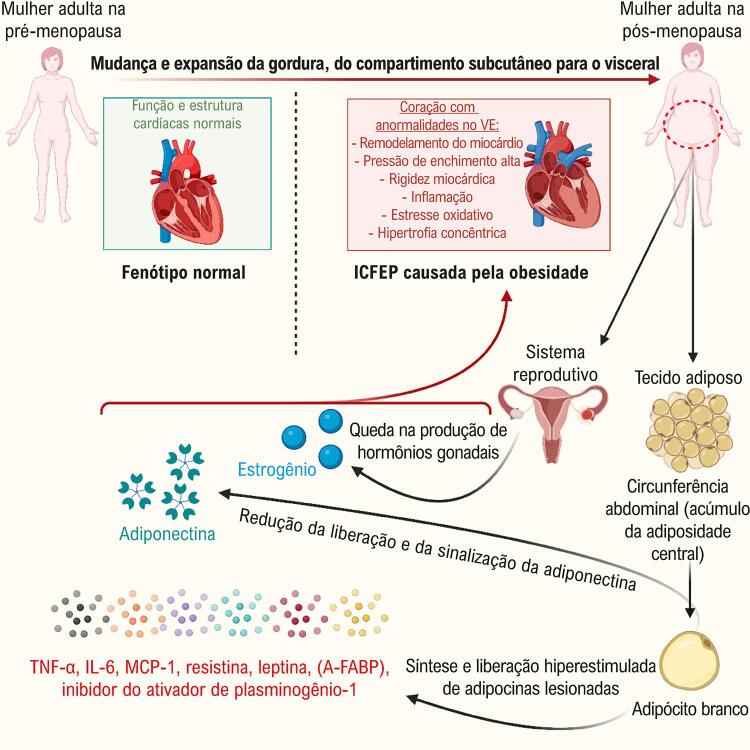
– *Diagrama esquemático mostrando o envolvimento de perda de estrogênio e obesidade na ICFEP. Uma expansão e mudança de gordura subcutânea para visceral ocorre em mulheres após a menopausa. A obesidade abdominal, definida pelo aumento da circunferência de cintura, é um grande fator de risco de desenvolvimento de ICFEP, que pode envolver uma tendência de aumento na síntese e na liberação de adipocinas, incluindo TNF-α, IL-6, MCP-1, resistina, leptina, lipocalina-2, e inibidor do ativador de plasminogênio-1. Essas adipocinas têm funções críticas em inflamação cardíaca, stress oxidativo, e disfunção metabólica. Por outro lado, a produção de adiponectina de adipócitos brancos, que tem efeitos benéficos na sensibilidade à insulina e função cardiovascular, é notadamente reduzida em indivíduos obesos. Anormalidades em adipocinas, além da perda de estrogênio, podem participar do desenvolvimento de ICFEP, induzindo a inflamação cardíaca e o stress oxidativo, e levando à hipertrofia cardíaca concêntrica, Remodelamento, rigidez, e disfunção diastólica. ICFEP, insuficiência cardíaca com fração de ejeção preservada; TNF-α, fator de necrose tumoral alfa; IL-6, interleucina 6; MCP-1, proteína quimiotática de monócitos; A-FABP, proteína transportadora de ácidos graxos de adipócitos.*

Não está claro se há diferenças entre os sexos nos efeitos da adiponectina na função e na estrutura cardíaca. Fontes-Carvalho et al.,^145^ relataram dados de um estudo populacional envolvendo indivíduos com 45 anos de idade ou mais sobre as associações dos níveis de leptina e adiponectina e a função diastólica do VE. Esses pesquisadores encontraram diferenças significativas entre os sexos para os níveis de leptina e adiponectina, e suas relações com a função diastólica. Níveis relativamente mais altos de leptina, associados a função diastólica piorada, foram relatados, especialmente entre mulheres, independentemente de idade ou hipertensão. É interessante notar que a adiponectina foi correlacionada a parâmetros da função diastólica.^145^ Ainda assim, é plausível postular um efeito específico do sexo para alterações de adiponectina na estrutura e na função miocárdica, já que as mulheres têm níveis de adiponectina sistêmica significativamente mais altos.^146^ Em dois estudos pequenos envolvendo pacientes que passam por angiografia coronária^147^ ou com IC,^142^ a redução dos níveis de adiponectina foi associada à piora da função diastólica. A Figura 1 resume os principais mecanismos envolvidos na perda de estrogênio e na obesidade no desenvolvimento de DDFVE e ICFEP.

## Conclusão

Em resumo, o estrogênio desempenha um papel essencial na regulação do peso corporal e da gordura corporal, e esse papel também pode ser a proteção do coração pré-menopausa da disfunção do VE. Em comparação com homens da mesma idade, as mulheres na pós-menopausa apresentaram aumento da rigidez ventricular e arterial, e têm mais probabilidade de desenvolver DDFVE e ICFEP subsequentes. Os níveis mais baixos de estrogênio depois da menopausa estão envolvidos nas mudanças da distribuição e no teor de gordura corporal, um fator que aumenta a incidência de DCV. Esta análise coletou as evidências recentes e esclareceu as rotas moleculares pelas quais o estrogênio desencadeia esses efeitos, e apresentou novas direções para pesquisa futura nessa área interessante do envelhecimento cardíaco específico dos sexos e da disfunção diastólica, que é um prenúncio de ICFEP.
