# Supplementary material for: Relações entre a Redução de Estrogênio, Obesidade e Insuficiência Cardíaca com Fração de Ejeção Preservada
Source: Arq Bras Cardiol. 2021 Sep 24;117(6):1191–201. [Article in Portuguese] doi: 10.36660/abc.20200855 (PMC8757160; doi:10.36660/abc.20200855)
Supplement: Translation

# Translation: Crossroads between Estrogen Loss, Obesity, and Heart Failure with Preserved Ejection Fraction

**Authors:** Allan Kardec Nogueira de Alencar, Hao Wang, Gláucia Maria Moraes de Oliveira, Xuming Sun, Gisele Zapata-Sudo, Leanne Groban

**Affiliations:** 1 Brazil Faculdade de Medicina de Petrópolis, Petrópolis, RJ - Brazil; 2 United States of America Wake Forest School of Medicine - Departments of Anesthesiology, Winston-Salem, North Carolina – United States of America; 3 Brazil Universidade Federal do Rio de Janeiro - Departamento de Clínica Médica, Faculdade de Medicina, Rio de Janeiro, RJ - Brazil; 4 Brazil Universidade Federal do Rio de Janeiro - Instituto de Ciências Biomédicas, Rio de Janeiro, RJ - Brazil; 5 Brazil Universidade Federal do Rio de Janeiro - Instituto de Cardiologia Edson Saad, Faculdade de Medicina, Rio de Janeiro, RJ - Brazil; 6 United States of America Wake Forest School of Medicine - Internal Medicine-Section of Molecular Medicine, Winston-Salem, North Carolina - United States of America

**Keywords:** Estrogens, Obesity, Heart Failure, Stroke Volume, Menopause, Adiposity, Overweight, Echocardiography/methods, Body Mass Index

## Abstract

The prevalence of obesity and heart failure with preserved ejection fraction (HFpEF) increases significantly in postmenopausal women. Although obesity is a risk factor for left ventricular diastolic dysfunction (LVDD), the mechanisms that link the cessation of ovarian hormone production, and particularly estrogens, to the development of obesity, LVDD, and HFpEF in aging females are unclear. Clinical, and epidemiologic studies show that postmenopausal women with abdominal obesity (defined by waist circumference) are at greater risk for developing HFpEF than men or women without abdominal obesity. The study presents a review of clinical data that support a mechanistic link between estrogen loss plus obesity and left ventricular remodeling with LVDD. It also seeks to discuss potential cell and molecular mechanisms for estrogen-mediated protection against adverse adipocyte cell types, tissue depots, function, and metabolism that may contribute to LVDD and HFpEF.

## Introduction

The prevalence of obesity is steadily increasing worldwide.^1^ Because obesity is associated with high mortality and the development of comorbid conditions, including diabetes mellitus and cardiovascular disease (CVD), it is one of the most difficult public health issues facing our society. This cluster of obesity-related comorbidities, whether directly or indirectly (e.g., side effect from anthracycline-formulated chemotherapy)^2^ often culminates in heart failure (HF).^3 - 6^

While obesity, defined as having a body mass index (BMI) > 30 kg/m^2^ , is an independent predictor of incident HF in the general population, evidence shows that even being overweight (BMI 25-29 kg/m^2^ ) carries an increased risk of HF.^7 - 10^

Several studies show that measures of central adiposity, such as waist circumference (WC), are superior to measures of global adiposity, such as weight and BMI, in estimating the risk of CVD.^11 - 16^ WC is independently associated with left ventricular diastolic dysfunction (LVDD), defined by echocardiographic parameters.^17^ Both LVDD and obesity are common factors that contribute to a heart failure with preserved ejection fraction (HFpEF) phenotype, and appear to be causally linked.^17 - 19^ Indeed, HF patients can have different phenotypes according to the morpho-functional characteristics of the disease.^20^ Briefly, HF patients are classified according to LV function; those with LV ejection fractions less than or equal to 40% fall into the category of heart failure with reduced ejection fraction (HFrEF), and patients with LV ejection fractions equal to or greater than 50% are deemed to have HFpEF. According to the American College of Cardiology and American Heart Association guidelines,^21^ there is also an intermediate or borderline group of patients who have ejection fractions between 41% and 49%, sometimes referred to as HFmEF. Moreover, a subset of patients with ejection fractions greater than 40% with HFpEF who previously had HFrEF is considered to be clinically distinct from those with persistently preserved or reduced ejection fractions. For purposes of this review, we have focused only on HFpEF, and, specifically, features of the “fat, female, and fatigued” obese HFpEF phenotype.^21^

For a narrative of the literature of all the clinical phenotypes of HFpEF, we refer the reader to the review by Silverman.^20^ Regardless of the biological phenotype, HFpEF is a heterogeneous clinical syndrome, including cardiomyocyte, extracellular matrix, vascular, and comorbidity-related pathophysiological mechanisms.^22^ It is characterized by reduced end-diastolic volume, left ventricular hypertrophy, and increased left atrial volume and left ventricular filling pressure. These pathophysiological abnormalities are associated with increased left ventricular stiffness, decreased left ventricular relaxation, cardiomyocyte hypertrophy, myocardial interstitial fibrosis, and reduced intramyocardial capillaries.^23 - 26^

Another important factor involved in the HFpEF phenotype is sex. HFpEF disproportionally affects more women (sex ratio of about 2:1) then men.^27 , 28^ The higher prevalence of HFpEF in elderly women^29^ appears related to the loss of ovarian hormones, primarily estrogens, that occur after the menopause.

Accordingly, in this review, we will explore pre-clinical and clinical data about the relationships between sex, “fatness”, including mechanisms of fat-induced cardiac impairment, specifically LVDD and HFpEF, and the cardioprotective effects of estrogen on fat metabolism in women.

### Link between “fatness”, sex, and HFpEF: clinical evidence

HF is a major problem that is increasing in scope. Despite recent therapeutic advances, morbidity and mortality after the onset of HF still remain substantial.^30^ Consequently, prevention of HF through the identification of the preclinical phases of the disease and the management of risk factors is a priority. Considering that 50 percent of all HF patients have HFpEF,^31^ the complex pathophysiology of this disease is still not fully understood, with no specific therapy available to improve patient outcomes. In this context, several studies have evaluated obesity as a risk factor for LV remodeling and subsequent HFpEF.^32 - 34^ In these studies, obesity has been consistently associated with LV stiffness and diastolic dysfunction, particularly in women.^17 , 35^

A community-based clinical study of 377 randomly selected participants older than 16 years of age assessed the independent contribution of indices of adiposity to variations in early-to-late (atrial) transmitral velocity (E/A), as determined by echocardiography. The main goal was to clarify why some previous studies failed to establish a contribution of obesity to LV diastolic function, while others demonstrated a relatively minor contribution. For each study participant, the independent relationship between adiposity and diastolic (E/A) or systolic (LV ejection fraction; LVEF) chamber functions were determined using multivariate linear regression analysis with adjustments for age, gender, conventional systolic or diastolic blood pressure measurements, and either LV mass index or relative wall thickness (calculated from echocardiography). Excessive central adiposity (WC), but not elevated BMI, was independently and inversely correlated with E/A; the investigators emphasized that WC might represent a progressive preclinical condition that contributes to obesity-induced diastolic HF.^36^ WC was second only to age and equivalent to blood pressure in the magnitude of effect on E/A. Findings from this study also suggested that, at a population level, LV mass and geometry play little or no role in the pathogenesis of obesity-induced LV diastolic abnormalities.^36^ Interestingly, there was no relationship between WC and LVEF (systolic dysfunction), confirming the findings of other researchers that identified WC as a risk factor for HFpEF.^7 , 9 , 17 , 37 - 43^ Finally, it is relevant to comment that the data reported by these authors were restricted to women as they have recruited a limited proportion of male participants.^36^ Additional data from the clinical study performed by Canepa et al.,^17^ in which the sample, from both genders, is part of the Baltimore Longitudinal Study of Aging (BLSA) propose that a possible pathophysiologic explanation for the association of central adiposity with worse cardiovascular outcomes is its relationship with LVDD. They found that central adiposity was strongly associated with LV dysfunction, particularly with impaired LV relaxation. The researchers also found that the effect of central adiposity on LVDD was independent of general adiposity and, surprisingly, this was more pronounced in women than in men. The gender-specific effect of central fat accumulation on LVDD was then graded using various echocardiographic parameters. The study confirmed their previous reports showing a negative correlation between the indices of adiposity and the E/A ratio; however, the authors also found that E/A alone was not enough to discriminate between subjects with normal or abnormal diastolic function and that mitral valve inflow velocities were also significantly influenced by the increase in preload, a condition frequently encountered in obese subjects. When these issues were normalized by combining tissue Doppler measures of mitral annular velocity, or e’ and dynamic (E/A) parameters, it was found that the E/e’ ratio was positively correlated with WC. Thus, this epidemiological study provided further evidence of the link between central obesity (WC) and the prevalence and development of LVDD, and that this link is influenced by sex.^17^

One limitation of cross-sectional studies is that they offer a snapshot of a single moment in time, and thus fail to capture changes that occur over time. The complex relationships between aging, sex, adiposity, and ventricular mechanisms were evaluated in a large longitudinal study conducted over a period of 4 years in 1,402 subjects, 45 years of age and older, who were randomly selected from a community-based population.^35^ It was found that weight gain during a 4-year period was associated with significant increases in LV diastolic stiffness in both men and women, but that it was more pronounced in women, indicating a sex difference in the biology of age-related ventricular stiffening. Furthermore, evaluating central obesity in women may help to identify a group at higher risk for incident HFpEF who might benefit from preventive treatment.^35^ Finally, the results of this longitudinal study confirmed the findings of cross-sectional investigations regarding the positive relationship between WC and echocardiographic measures of diastolic dysfunction (e.g., E/e’ ratio).

How changes in fat tissue depot might influence the link between obesity and impairments in cardiac function and remodeling, particularly among women, are important to consider. While female sex hormones are believed to cause fat to be stored in the buttocks, thighs, and hips of women, which may be essential for normal reproduction purposes, menopause-related changes in body fat distribution may partially explain the increased risk of cardiovascular and metabolic disease during the postmenopausal years.^44 - 46^ In 2011, Wehr et al.,^47^ published results from a longitudinal study of gender-specific differences in the relationship between the lipid accumulation product, which is calculated from WC, and cardiovascular mortality, as well as the presence of type 2 diabetes. The study included 2,279 men and 875 postmenopausal women, with a median follow-up of 77 years. Lipid accumulation product levels were independently associated with congestive HF mortality in all postmenopausal women and with all-cause mortality in diabetic postmenopausal women, but not in men. These data not only support the concept that fat redistribution after estrogen loss may contribute to cardiovascular disease progression, but also endorse an inexpensive and simple risk biomarker, namely, lipid accumulation product, which could identify postmenopausal women at higher cardiovascular risk.^47^

### Treatment modalities for weight loss: an evidence-based approach to measure outcomes in patients with HFpEF

Given clinical evidence for crosstalk between the heart and “fatness”, with respect to female sex-specific HFpEF, weight reduction or maintenance of ideal body weight is one preventive approach to mitigate age- and estrogen-loss related changes in ventricular structure and function. Essential treatments for weight reduction include changes in eating habits to reduced-calorie and low-fat diets, increased physical activity or exercise, and other behavioral modification strategies, such as self-monitoring (e.g., daily record keeping of food intake and exercise), stimulus control (e.g., avoiding triggers that prompt eating), and problem solving (e.g., identifying barriers and ways to overcome them). Additionally, bariatric surgery is another effective strategy to treat severely obese patients. Thus, it is important to review the literature addressing the effects of different weight loss strategies on the cardiovascular outcomes in patients with LVDD and HFpEF.

Observational studies suggest that overweight or mildly-to-moderately obese patients with HFpEF survive longer than those who are normal-weight.^18 , 48^ However, Kitzman et al.^49^ recently reported that 20 weeks of caloric restriction combined with aerobic exercise training among obese older patients with HFpEF reduced their body weight with additive improvements in exercise capacity, defined by peak V0_2_. Even so, only caloric restriction resulted in decreases in LV mass and relative wall thickness, along with an inkling for improvements in diastolic function, as per the increase in E/A observed in this treatment arm, without effecting resting cardiac function, depicted by ejection fraction or Doppler-derived cardiac output.^49^ It was further reported that no changes in magnetic resonance imaging (MRI) measures of epicardial or pericardial fat were observed across treatments, but that there were significant reductions in thigh and abdominal subcutaneous and visceral fat depots in the diet only group.^49^ While these findings favor a role for weight reduction strategies, along with exercise, to improve the detriments in exercise capacity and maximal oxygen consumption associated with HFpEF among obese patients, they also support the concept that extra-cardiac mechanisms are uniquely involved in the pathogenesis of HFpEF.^50 , 51^

Another weight reduction strategy that is conducive to evaluating the link between “fatness”, LVDD, and HFpEF is bariatric surgery. Indeed, morbidly obese patients who commonly present hemodynamic and cardiac morphometric characteristics, such as elevations in cardiac preload and afterload and increases in LV chamber and wall dimensions, which contribute to myocardial stiffness and impairments in myocardial relaxation.^52^ Multiple clinical studies^52 - 55^ and one systemic review with a meta-analysis^56^ reported the benefits of bariatric surgery with subsequent weight loss on echocardiographic and MRI measures of LV structure, including substantial reductions in LV wall thickness and mass and diastolic function. Along with upgrades in diastolic function, others found that extensive weight loss after bariatric surgery also led to favorable changes in muscle metabolism^57^ and characteristics of arterial elasticity.^58 , 59^

### Link between cardiac-specific regional adiposity, LVDD, and HFpEF

In addition to peripheral and total body fat linked to LVDD, the potential role of cardiac-specific regional adiposity (e.g. pericardial and epicardial fat depots) in the disease process should not be ignored.^60^ Pericardial and epicardial fat, commonly found in obese and overweight patients, are considered ectopic adipose depots that can induce a lipotoxic state in close proximity to cardiac muscle and coronary arteries.^61 , 62^ Moreover, we know that metabolic syndrome, a common condition among obese and overweight patient,^63^ is associated with increased adipose tissue volume around the heart,^60^ particularly the accumulation of epicardial fat,^64^ which is significantly linked to adverse cardiovascular events,^65 - 69^ including HFpEF.^61 , 70 , 71^ The direct correlation between these local fat depots and LVDD can be explained, in part, by paracrine processes, whereby proinflammatory cytokines and other damaging mediators (e.g. TNF-α and IL-6),^72^ collectively called adipokines, are released from the local adipose repositories.^61 , 72 - 74^

Distinguishing between the two fat depots and their respective link to LVDD may be important both anatomically and biochemically. For instance, epicardial fat is located between the outer wall of the heart muscle and the visceral layer of pericardium,^64^ and its proximity to the myocardium is significant in that both tissue layers share the same blood microcirculation, the coronary arteries.^64^ Potential interactions can be elicited when dysfunctional adipocytes from cardiac fat depots release proinflammatory adipokines into the microcirculation,^37^ which in turn can interact with cardiomyocytes and cardiac fibroblasts. These cells independently respond to adipokines, which contribute to the pathologic process of myocardial fibrosis,^75^ thereby leading to myocardial remodeling, via low grade inflammation and fibrotic processes, which can intensify LV hypertrophy, wall stiffness, and LVDD progression.^76 - 79^ Pericardial fat, which can be more specifically referred to as *para* -cardial fat or intrathoracic fat,^80^ is that fat which is deposited outside the parietal pericardium. This fat depot originates from primitive thoracic mesenchyme and is supplied by noncoronary sources. While increases in paracardial fat volume in HFpEF have been reported to induce a mechanical compressive-like load on the myocardium, which impairs LV filling,^81^ paracrine processes have also been noted. Excessive pericardial adiposity contains high levels of proinflammatory mediators that, when released from adipocytes, promote a collagen turnover, thus leading to myocardial stiffness, impaired lusitropism and subsequent LVDD.^82^ Indeed, Konishi et al.,^61^ reported that a high volume of pericardial fat was significantly correlated with Doppler-derived increases in filling pressure, or E/e’, in HFpEF patients. Moreover, studies have documented a strong potential for epicardial adiposity to be associated with poor prognosis in obese or overweight patients with LVDD and HFpEF.^71 , 83 - 85^

Given the link between local cardiac fat depots and adverse cardiovascular health, weight reduction strategies should be strongly considered among the armamentarium in the management of the obese LVDD patient. Interestingly, in obese postmenopausal women with HFpEF, Brinkley et al.,^86^ showed that caloric restriction, aerobic exercise, or combination therapy significantly reduced body weight and pericardial fat, and the changes in pericardial fat were inversely correlated with cardiorespiratory fitness defined by VO_2_max. Certainly, future therapies targeting low-grade inflammatory processes arising from epi- and pericardial fat depots could also limit the progression of LVDD.

### Link between estrogen and fat-induced cardiovascular risk

The high predilection of HFpEF among older women compared to older men with HF is well accepted.^27 , 28^ The role that differences in adipocyte distribution among men and woman might have with respect to this sex-specific differential in HF prevalence is new and still coming together. Indeed, women have more body fat than men, but in contrast to the adverse metabolic consequences of central obesity that is typical of men, the pear-shaped, or gluteal-femoral body fat subcutaneous distribution of many women is associated with lower cardiometabolic risk.^87 , 88^ However, with advancing age there is a general shift and expansion of fat from the subcutaneous to visceral compartment.^87 - 89^ In aging males this means expansion of abdominal visceral adiposity, while in aging females this involves a redistribution of fat from the subcutaneous gluteal-femoral compartment to the visceral-abdominal compartment.^87 - 89^ In both cases, cardiovascular disease risk increases with age-related abdominal compartment expansion of visceral fat.^89 , 90^

As outlined in the introduction, loss of gonadal hormones in older women seems to represent a component associated with increased risk for developing HFpEF. Since women are less likely to develop CVD before menopause,^91^ the production of ovarian estrogen appears to protect against HF.^92 , 93^ Consistently, there are several reports confirming the beneficial effects of estrogen in the cardiovascular system.^94 - 96^

To understand the specific role of gonadal hormones in the expansion of age-related visceral fatness in women and, in turn, its potential influence on diastolic function, a brief review of gonadal hormones, particularly the estrogens and their receptors, is first warranted. The three naturally occurring estrogens in women are estrone (E1), estradiol (E2), and estriol (E3). A fourth form of estrogen, estetrol (E4), is produced only during pregnancy. All these different estrogen forms are synthesized from androgens.^97^ For simplicity, we will use the term estrogen, to include all forms.

Estrogen binds to multiple receptors, including classical nuclear estrogen receptors (ERs), ERα, and ERβ, and a G protein-coupled receptor, GPER.^98^ The ERs signal not only through a “classical” regulation of gene transcription, but also by activating a “non-nuclear” signaling pathway.^94 , 99^ Accumulating findings have been well described and reviewed in the literature, concerning the roles triggered by ERs in maintaining the homeostasis of the cardiovascular system.^99 - 101^

Estrogen directly regulates adiposity distributions through estrogen receptors. In the premenopausal state, subcutaneous fat has relatively more estrogen and progesterone receptors than androgen receptors, whereas visceral fat has higher levels of androgen receptors.^102^ With menopause, the fall of estrogen leads to estrogen receptors on subcutaneous fat to be inactivated, while the androgen receptors on visceral fat become relatively activated, thereby contributing to the inverse relationship between estrogen levels and visceral fat.^103 , 104^ Likewise, in estrogen deficient rodent models induced by ovariectomy, the observed increase in body weight is mainly due to gains in visceral fat.^105^ Estrogenic protection can further be seen upon the systemic administration of estrogen in OVX models whereby the body fat distribution mirrors that of gonad-intact counterparts.^106^

The specific roles may offset the classical steroid receptors ERα and ERβ in the context of fat one to another. In a recent study by Zidon et al.,^107^ gonad intact ERα KO mice were found to be 25% heavier with reduced energy expenditure compared to age-matched gonad intact wild-type and ERβ KO mice.^107^ Furthermore, following OVX, αKO mice did not increase body weight or exhibit more pronounced insulin resistance, whereas WT and βKO mice did, suggesting that the loss of signaling through ERα facilitates OVX-induced metabolic dysfunction. These new data further suggest that following estrogen deficiency, ERβ may mediate protective metabolic benefits.^107^ This contradicts previous preclinical reports showing that the two classical ERs on adipose tissue regulate fat reciprocally.^108 - 110^ Despite this discrepancy, the linkage between polymorphisms in the structure of both classical ERs and increased CV risk among postmenopausal women corroborate the important role of ER isoforms in the regulation of adiposity, metabolic derangement, and cardiovascular risk.^111 - 113^

### Fat-derived adipokines and roles in cardiovascular disease risk

The main role of brown fat, mainly located around the neck and large blood vessels of the thorax, is to generate heat by “uncoupling” the respiratory chain of oxidative phosphorylation within mitochondria.^114^ White visceral fat (abdominal fat) is mainly involved in a complex and multidirectional network of autocrine, paracrine, and endocrine signaling that crosstalk between organs and tissues. It is the white fat that mainly participates in the pathogenesis of metabolic diseases, such as type 2 diabetes mellitus, insulin resistance, hypertension, coronary heart disease, stroke, and HF.^17 , 115 , 116^ It is currently well accepted that adipose tissue is an active endocrine organ that secretes heterogeneous bioactive factors called adipokines,^115^ including cytokines and chemokines, vasoactive and coagulation factors, regulators of lipoprotein metabolism, and proteins, such as adiponectin and leptin.^115^

In obesity, enlargement of adipose tissue mass has been linked to a dysregulation of adipokine secretion and the related tissue inflammation, which represents a critical pathogenic link between obesity and the development of cardiometabolic diseases.^117^ In obese individuals, adipose tissue is infiltrated with activated macrophages and several other types of inflammatory cells, leading to an augmented production of proinflammatory adipokines, such as TNF-α, IL-6, monocyte chemoattractant protein (MCP)-1, resistin, leptin, lipocalin-2, adipocyte fatty acid binding protein (A-FABP), and plasminogen activator inhibitor-1.^116^ These inflammatory factors are key components of the “adipo-cardiovascular axis” that mediates crosstalk between adipose tissue and the CV system.

Among the various adipose depots, perivascular adipose tissue is an important contributor to vascular inflammation because of its proximity to the blood vessel wall and its pronounced proinflammatory properties. Proinflammatory cytokines/adipokines released from other major adipose tissue depots, such as subcutaneous and abdominal fat, may further contribute to vascular inflammation by virtue of their endocrine actions.^116^ These findings explain, in part, why WC may be considered a surrogate biomarker of CVD risk.

Adiponectin is one of the most abundant adipokines secreted by adipocytes, accounting for 0.01% of the total plasma protein content in humans.^118^ The production of adiponectin from white adipocytes, which exerts beneficial effects on insulin sensitivity and cardiovascular function, is markedly reduced in obese individuals.^116 , 119 , 120^ Epidemiological studies show that low circulating adiponectin levels, particularly the high molecular weight form, is a risk factor for type 2 diabetes, hypertension, atherosclerosis, and myocardial infarction.^116^

#### Adiponectin and adiponectin receptors

The relationship between obesity and LVDD may be linked to adiponectin and adiponectin receptors. The full-length adiponectin consists of 247 amino acid residues, assembled into an *N* -terminal hypervariable region followed by a conserved collagenous domain of 22 Gly-Xaa-Yaa repeats and a *C* -terminal C1q-like globular domain.^119^ In human and mouse plasma, adiponectin is present in three major oligomeric forms.^119 , 121 , 122^ The monomeric form has never been detected under native conditions. The basic unit of oligomeric adiponectin is a homotrimer called low-molecular-weight (LMW) adiponectin.^119 , 123 , 124^ Two subunits of the adiponectin trimer are linked by a disulfide bond via cysteine residues in the collagen-like domain, which forms a hexamer termed middle-molecular-weight (MMW) adiponectin. This hexamer provides the building block for the formation of the bouquet-like high-molecular-weight (HMW) adiponectin comprised of 12-18 hexamers.

Post-translational modifications to the adiponectin protein are required for the intracellular assembly of the HMW oligomeric complex in adipocytes.^125^ Different oligomeric forms of adiponectin act on different targets and possess distinct biological functions.^119^

The two main adiponectin receptors (AdipoRs), AdipoR1 and AdipoR2, are structurally and functionally distinct from classic G-protein-coupled receptors.^126^ AdipoR1 is expressed ubiquitously, whereas AdipoR2 is expressed most abundantly in the liver.^126^ Both AdipoR1 and AdipoR2 are expressed in cardiac cells,^127^ but the exact roles of these two receptors in the antioxidative/nitrative stress and anti-inflammatory actions in cardiomyocytes remain unclear.

Although adipocytes are the major contributors to plasma adiponectin, adiponectin is also expressed in cardiomyocytes,^127^ and cardiomyocyte-derived adiponectin is biologically active in protecting cells against ischemic injury via paracrine/autocrine activation of cardiac AdipoRs in mice.^128^ In patients with dilated cardiomyopathy, cardiac adiponectin expression is downregulated.^129^

#### Adiponectin and LVDD

In addition to AdipoRs, T-cadherin has also been suggested as a potential receptor for adiponectin,^130^ and it is highly expressed in the heart, smooth muscle, and endothelium, representing the main target of adiponectin in the cardiovascular system.^131 , 132^ T-cadherin is anchored at the cell surface by glycosyl phosphatidylinositol, and it plays an indispensable role in adiponectin-induced cardioprotection in mice,^133^ acting as a physiological adiponectin-binding receptor that enables the association of this adipokine with cardiac tissue.^133^

As low levels of adiponectin have been linked to obesity-related cardiometabolic complications, its role in the maintenance of cardiac health should not be ignored.^120 , 134 , 135^ Preclinical data show that adiponectin can attenuate or prevent the progression of LVDD to HFpEF.^120 , 136 , 137^ In a mouse model of aldosterone-induced HFpEF, Sam et al.^137^ showed that a lack of adiponectin was associated with increased systolic blood pressure, LV remodeling, diastolic dysfunction, and pulmonary congestion, while the chronic hyperadiponectinemia in transgenic mice overexpressing adiponectin, reported by Tanaka et al.,^120^ ameliorated aldosterone-induced LV hypertrophy, diastolic dysfunction, and lung congestion, regardless of changes in blood pressure. The early filling-to-early mitral annular descent ratio, or E/e’, which was increased in the aldosterone-induced HFpEF mice and indicative of elevated filling pressures,^137^ which was significantly attenuated in adiponectin transgenic mice. Tanaka et al.,^120^ also found that adiponectin overexpression decreased myocardial oxidative stress and calcium handling by preserving protein kinase A (PKA)-dependent phosphorylation of phospholamban. Moreover, adiponectin replacement in adiponectin knock-out mice, attenuated transmitral Doppler indices of pseudonormalization, indicative of impaired LV compliance.^120^ Taken together, these preclinical findings suggest that adiponectin may have a therapeutic potential in the management of LVDD and HFpEF, via direct actions on the heart.

Our observation that circulating adiponectin was not associated with LV fractional shortening is consistent with existing literature, which suggests that adiponectin acts primarily as an inhibitor of cardiac hypertrophy.

Clinical studies also support an association between adiponectin and cardiac function and structure.^129 , 138^ In a community-based cohort study, McMannus et al.^139^ showed that relatively higher plasma levels of adiponectin associated with reduced LV mass, suggesting a cardioprotective effect. There are additional clinical data addressing the cardioprotective roles of adiponectin on the heart.^140 - 142^ For instance, Francisco et al.^143^ comprehensively reviewed the relevance of adiponectin signaling for the prevention of obesity related diastolic dysfunction with an emphasis on its role in limiting myocardial hypertrophy, cardiac fibrosis, nitrative and oxidative stress, atherosclerosis, and inflammation.

As there are several studies showing adiponectin’s modulatory role in the maintenance of diastolic function, it is worth mentioning that other investigators report no relationship to fat-associated LVDD. Sawada et al.^144^ found that while plasma adiponectin levels significantly decreased with increased visceral adiposity in the general population, the association between adiponectin and diastolic function was not independent of fat. In other words, decreased circulating levels of adiponectin did not appear to have a central role in the association between visceral adiposity and LVDD. As this work was performed in a population with normal or grade 1 LVDD, subjects with more than moderate LVDD were not included. These authors suggested that a larger-scale study, including patients with moderate or severe LVDD are needed to confirm findings.

#### Sex differences, adiponectin, and cardiac function

Whether there are sex differences in the effects of adiponectin on cardiac function and structure is not clear. Fontes-Carvalho et al.^145^ reported data from a population-base study involving individuals aged 45 years and older on associations of leptin and adiponectin levels and LV diastolic function. These investigators found significant sex differences for both leptin and adiponectin levels and their relationships with diastolic function. Relatively higher levels of leptin were associated with worse diastolic function, particularly among women, and this was regardless of age and hypertension. Interestingly, adiponectin did not correlate with diastolic function parameters.^145^ Even so, it is plausible to postulate a sex-specific effect for adiponectin on changes in myocardial structure and function, as women have significantly higher systemic adiponectin levels.^146^ In two small studies involving patients undergoing coronary angiography^147^ or with HF,^142^ decreased adiponectin levels were associated with worse diastolic function. Figure 1 summarizes the main mechanisms involved in estrogen loss and obesity in the development of LVDD and HFpEF.

**Figure 1:** – *A schematic diagram showing the involvement of estrogen loss and obesity in HFpEF. An expansion and a shift of subcutaneous to visceral fat occurs in women after menopause. Abdominal obesity, defined by increased waist circumference, is a major risk factor for the development of HFpEF, which may involve an uptick in the synthesis and release of adipokines, including TNF-α, IL-6, MCP-1, resistin, leptin, lipocalin-2, and plasminogen activator inhibitor-1. These adipokines play critical roles in cardiac inflammation, oxidative stress, and impaired metabolism. On the other hand, the production of adiponectin from white adipocytes, which exerts beneficial effects on insulin sensitivity and cardiovascular function, is markedly reduced in obese individuals. Abnormalities in adipokines, in addition to estrogen loss, might participate in the development of HFpEF by inducing cardiac inflammation and oxidative stress, and finally leading to concentric cardiac hypertrophy, remodeling, stiffness, and diastolic dysfunction. HFpEF, heart failure with preserved ejection fraction; TNF-α, tumor necrosis factor alpha; IL-6, interleukin 6; MCP- 1, monocyte chemoattractant protein; A-FABP, adipocyte fatty acid binding protein* .

## Conclusion

In summary, estrogen plays a major role in the regulation of body weight and body fat, and that role may also protect the premenopausal heart from LV dysfunction. Compared with age-matched men, postmenopausal women display increased ventricular and arterial stiffness, and are more likely to develop LVDD and subsequent HFpEF. The lower estrogen levels after menopause are involved in changes in body fat distribution and content, a factor that increases the incidence of CVD. This review collected the recent evidence and clarified the molecular routes by which estrogen triggers these effects and provides new directions for future research in this exciting area of sex-specific cardiac aging and diastolic dysfunction, which is a harbinger for HFpEF.
